# Imprinted DNA methylation reconstituted at a non-imprinted locus

**DOI:** 10.1186/s13072-016-0094-0

**Published:** 2016-09-22

**Authors:** David H. Taylor, Chelsea M. McLean, Warren L. Wu, Alex B. Wang, Paul D. Soloway

**Affiliations:** 1 Field of Genetics, Genomics, and Development, College of Agriculture and Life Sciences, Cornell University, Ithaca, NY USA; 2 Department of Molecular Oncology, The Netherlands Cancer Institute, Plesmanlaan, Amsterdam, The Netherlands; 3 Division of Nutritional Sciences, College of Agriculture and Life Sciences, Cornell University, Ithaca, NY USA; 4 Field of Biochemistry, Molecular and Cell Biology, College of Agriculture and Life Sciences, Cornell University, Ithaca, NY USA

**Keywords:** Imprinting control region, *Rasgrf1*, *cis* element, DNA methylation, CTCF, *Wnt1*

## Abstract

**Background:**

In mammals, tight regulation of cytosine methylation is required for embryonic development and cellular differentiation. The *trans*-acting DNA methyltransferases that catalyze this modification have been identified and characterized; however, these proteins lack sequence specificity, leaving the mechanism of targeting unknown. A *cis*-acting regulator within the *Rasgrf1* imprinting control region (ICR) is necessary for establishment and maintenance of local imprinted methylation. Here, we investigate whether 3-kb of sequence from the *Rasgrf1* ICR is sufficient to direct appropriate imprinted methylation and target gene expression patterns when ectopically inserted at the *Wnt1* locus.

**Results:**

The *Rasgrf1* ICR at *Wnt1* lacked somatic methylation when maternally transmitted and was fully methylated upon paternal transmission, consistent with its behavior at the *Rasgrf1* locus. It was unmethylated in the female germline and was enriched for methylation in the male germline, though not to the levels seen at the endogenous *Rasgrf1* allele. *Wnt1* expression was not imprinted by the ectopic ICR, likely due to additional sequences being required for this function.

**Conclusions:**

We have identified sequences that are sufficient for partial establishment and full maintenance of the imprinted DNA methylation patterns. Because full somatic methylation can occur without full gametic methylation, we infer that somatic methylation of the *Rasgrf1* ICR is not simply a consequence of maintained gametic methylation.

**Electronic supplementary material:**

The online version of this article (doi:10.1186/s13072-016-0094-0) contains supplementary material, which is available to authorized users.

## Background

Cytosine methylation (5mC) is vital for the regulation of development and other essential processes such as X-chromosome inactivation, genomic imprinting, transposon silencing, and terminal differentiation. 5mC is usually associated with transcriptional repression, but is known to be a complex and dynamically regulated modification. Research into this regulation has included descriptive studies such as methylome mapping [1, 2] or functional studies of the proteins that regulate methylation in *trans*, such as DNMT1 [3], and isoforms of DNMT3 [4–6]. Few studies, however, have yielded much information about the *cis*-acting DNA sequences that target these *trans*-acting proteins to the DNA in a locus-, tissue-, or time-specific manner. These few informative studies have used imprinted loci, which undergo highly regulated parental-specific, monoallelic methylation and expression. *Cis*-acting regulators of 5mC have been identified at *Igf2r* [7], *Snrpn* [8, 9], *H19* [10–14], and *Rasgrf1* [15–17], and in some cases, their mechanisms of action have been elaborated.

**Fig. 1:**
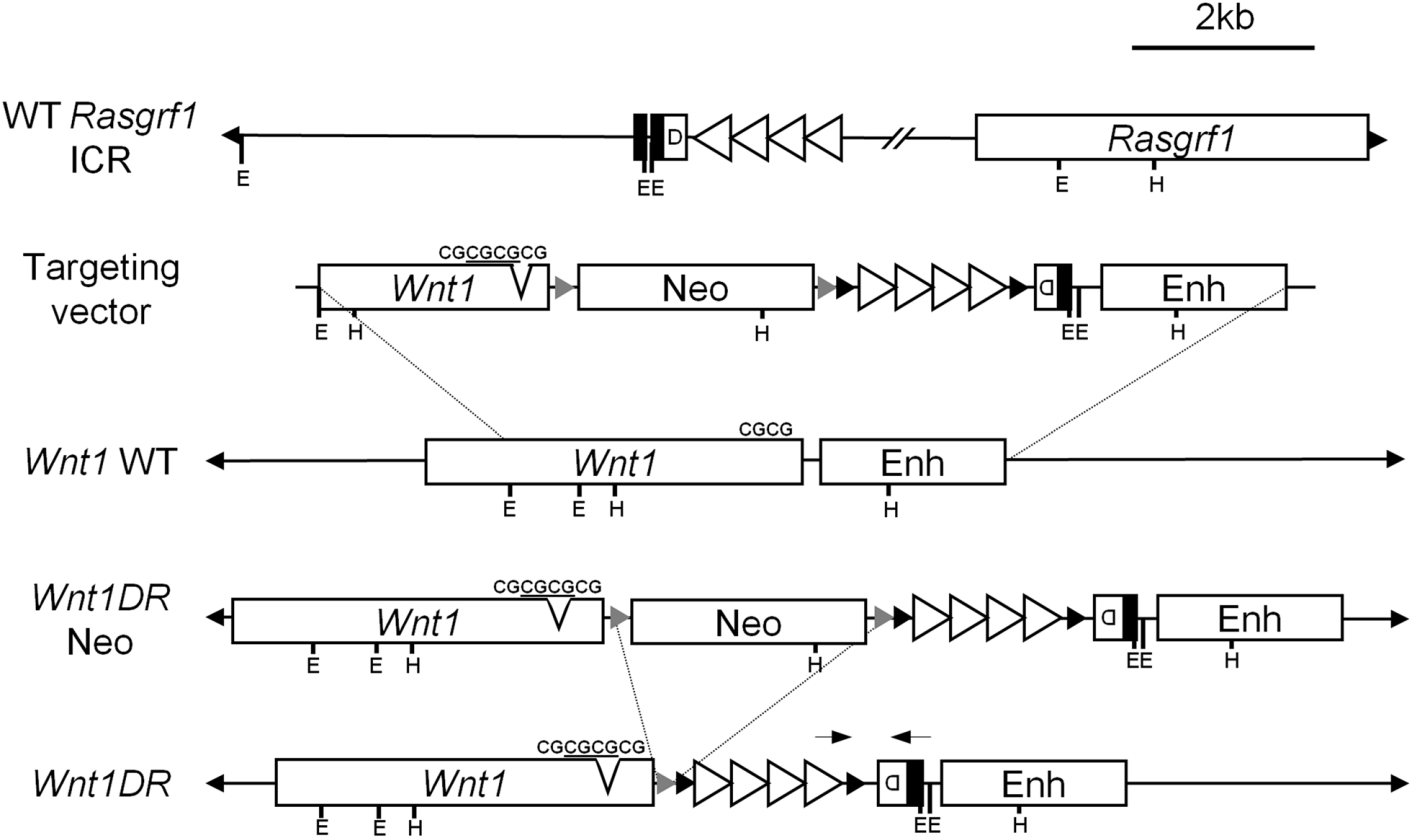
Development of the *Wnt1DR* mutant allele. At the wild-type (WT) *Rasgrf1* locus, the ICR includes the repeat region (large *white triangles*) directly adjacent to the DMD (D), and sites 1 and 2 (*vertical black bars*) from which secondary piRNAs are processed. The *Wnt1DR* targeting construct contains an FRT-flanked (*small gray triangles*) neomycin resistance cassette (Neo), loxP-flanked (*small black triangle*) repeats, the DMD, and a CGCG insertion in the *Wnt1* 3′ UTR (divot in UTR, *underlined sequence* is *Wnt1DR* specific) for allele-specific expression analysis. Primers for detecting *Wnt1DR*-specific methylation are denoted with* black arrows*. HindIII (H) and EcoRI (E) sites

The *Rasgrf1* ICR that lies 30-kb upstream of the paternally expressed *Rasgrf1* gene has two components: a differentially methylated domain (DMD) and an adjacent series of 41-nt tandem repeats, 2 kb in length [15] (Fig. 1). The repetitive element, which is required for proper methylation establishment [15] and maintenance after fertilization [18], acts as a promoter for a piRNA-targeted noncoding RNA (pitRNA) that is transcribed across the DMD in e16.5 testes [16]. piRNAs normally silence transposable elements in the male germline; however, a subset of these primary piRNAs interact with two loci within the pitRNA, called sites 1 and 2. The pitRNA is subsequently processed to secondary piRNAs, and de novo methylation of the ICR depends on piRNA pathway components MITOPLD and MILI.

Hypothesizing that repeat-mediated pitRNA transcription and the piRNA binding sites are sufficient to recapitulate imprinting, Park et al. [17] showed that proper sex-specific establishment of germline 5mC in transgenic reporter mice where the ICR was inserted between the γ-globin promoter and its enhancer. Proper somatic maintenance of 5mC was observed in subsequent generations if the construct was maternally transmitted; however, this construct was not sufficient to maintain an unmethylated maternal DMD in somatic tissue if it passed through the paternal germline. Failure of maintenance was likely due to the inappropriate methylation of a neomycin resistance selectable marker (neo) within the transgene which spread onto the adjacent ICR. In support of this, methylation analysis showed somatic methylation of the transgenic neo cassette upon maternal allele transmission, even if the transgenic DMD was unmethylated. These results confounded efforts to define the minimal sequences sufficient for *Rasgrf1* imprinting.

In order to define the features of the *Rasgrf1* ICR that are sufficient for imprinting control without confounding effects of sequences from other species or ambiguity of transgene insert sites, we targeted the ICR to the non-imprinted *Wnt1* locus in mouse. Here, we show that site 1 and the repeats are sufficient to establish wild-type somatic imprinted methylation patterns at this ectopic locus. In keeping with previous studies, however, the imprinted expression patterns could not be recapitulated, possibly due to the complexity of the local chromatin context- or tissue-specific decreases in CTCF binding.

## Methods

### Generation of targeted mice

The *Wnt1*^*tm1pds*^ vector was assembled using genomic clones from *Rasgrf1*, and PCR products from *Wnt1*. It included a loxP site at the *Wnt1* DMD that enabled us to distinguish it from the endogenous *Rasgrf1* DMD, and which we previously showed did not interfere with DNA methylation at *Rasgrf1* [18]. The vector also included a 4-nt insertion at a *Mlu*I site in the *Wnt1* 3′ UTR, creating a *Bss*HII site that enabled us to design allele-specific primers that distinguished transcripts from the endogenous and mutated *Wnt1* locus. The vector was linearized with *ZraI*, electroporated into v6.5 ES cells, and placed under 200ug/ml G418 selection. Correct integration was verified by Southern blot after *NdeI* digestion using both a 5′ (amplified by PDS497 5′-GAAGTGGGGCACATCATT and PDS492 5′-CATTTGCACTCTCGCACA) and 3′ (amplified by PDS349 5′-AATATGCCTGACGCACCTTC and PDS 350 5′-CACTTCTCTCTGGGCCTCAC) external probes. The neo resistance cassette was removed using transient lipofection of the pCAGGS-flpe-puro plasmid (Addgene #20733). Cells were then microinjected into C2J blastocysts (Jax Stock No: 000058) and subsequently implanted into FVB pseudopregnant females. Germline transmission was verified by an internal PCR (PDS288 5′-TTACCCAGCTTCTCATAGGCGC and PDS1749 5′-CTGCAATTTCTGCCATCATC). Mice were then bred into the C57BL/6 background. The mutated *Wnt1* allele is referred to as *Wnt1*DR.

### Swim-up assay

Swim-up assays were adapted from standard human protocols [19]. Briefly, sperm were isolated from cauda epididymis, washed in 1 ml cell culture media supplemented with BSA, and pelleted gently at 300×*g* for 10 min, after which the supernatant was discarded. After gently adding 300 ul of fresh media, the pellet was incubated for 60 min at 30 °C to allow motile sperm to enter the supernatant. The supernatant was then gently separated from the pellet, and both samples were processed for DNA methylation analysis.

### Methylation

DNA for methylation analysis was bisulfite converted using the Zymo Methylation-Lightning kit (#D5030) and amplified using allele-specific PCR for the *Wnt1DR* DMD (PDS405 5′-GTCGTTAAAGATAGTTTAGATATGG and PDS2172-2175 5′-ACAACRAAATACRACAATCACTAATAC) for 40 cycles. Oocyte DNA from 50 oocytes was pooled with salmon sperm as a carrier before conversion. Because of the exceedingly small amount of oocytes template, a nested approach was followed (PDS271 5′-GGAATTTTGGGGATTTTTTAGAGAGTTTATAAAGT and PDS2172-2175 5′-ACAACRAAATACRACAATCACTAATAC) for 15 additional cycles to improve yield. The bisulfite PCR products were purified (Qiagen PCR purification kit #28104), end-polished (End-IT kit #ER0720) for 45 min, A-tailed (NEB Klenow exo- # M0212L) for 50 min, and ligated with TruSeq adapters. This product was subjected to 10 rounds of amplification with barcode-specific primers (PDS2700 5′-AATGATACGGCGACCACCGA and PDS2701 5′-CAAGCAGAAGACGGCATACGA). Ampure beads (Agencourt #A63880) were used to clean up between steps. The ligated product was then gel purified (Qiagen Gel extraction kit # 28704) and quantified with the Qubit DNA HS kit (ThermoFisher # Q32851) before pooling and sequencing using MiSeq. The resulting reads were quality controlled and trimmed, enabling the analysis of at least 1,000 high-quality sequences per sample (Summary Table of read results in Additional file 1) using a local installation of the Quantitative Methylation Analysis online software (QUMA http://quma.cdb.riken.jp/).

### Expression analysis

cDNA was created via random hexamer reverse transcription from Trizol-extracted RNA derived from e9.5 mouse brains. *Wnt1DR* expression was measured by qRT-PCR and SYBR-green using primers specific to a four-nucleotide polymorphism in the 3′ UTR of the *Wnt1DR* allele (PDS2037 5′-CTGCCTCCTCATCACTGTGTAAATA and PDS2039 5′-ATAACCGAACGCGCGCGTG). Allele-specific expression was normalized to total *Wnt1* expression (PDS2037 5′-CTGCCTCCTCATCACTGTGTAAATA and PDS2038 5′-CTGGAACCCAGCACAATAAATAGTTT).

### Chromatin immunoprecipitation (ChIP)

CTCF ChIP was conducted using standard protocols. Briefly, brains from e9.5 mice carrying the *Wnt1DR* allele, transmitted either maternally or paternally, were finely minced smaller than 1 mm^3^, fixed for 10 min in a 1.1 % formaldehyde solution, quenched with 2.5 M glycine for 5 min at a final concentration of 125 mM, disaggregated using 30 strokes of a Dounce homogenizer, sonicated with the Covaris S2 Acoustic Disrupter (duty cycle = 5 %, intensity = 2, cycles/burst = 200, cycle time = 3′ ON/60″ OFF, 3 cycles), and purified using 4 ul of CTCF antibody [Kim et al. Cell. 128: 1231–45. (2007)] or IgG (Upstate #12-371) on protein A beads (Life Technologies #10003D) with multiple high stringency washes. Mouse embryonic fibroblasts (MEFs) were grown on 150-mm plates (~10 million/experiment) and subjected to the same ChIP protocol, except without a Dounce homogenization step. CTCF binding was verified using qPCR with primers specific to the *H19* ICR as a positive control (PDS2825 5′-ATAGCCAAATCTGCACAGCG and PDS2826 5′-CATAAGGGTCATGGGGTGGT), an intergenic region as a negative control (from chr. 9 89646705–89646598, numbering based on NCBI37/mm9; PDS2827 5′-AAGAAGCTGCTGAAACACCG and PDS2828 5′-TGCTGGGTGGTACTGGTATG), and at the *Wnt1DR* DMD (PDS2846 5′-CGAAGTTATATCGATAAGCTGCTG and PDS2847 5′-CTACCGCTGCGCTACAACTA).

## Results

To test the sufficiency of the *Rasgrf1* ICR to impart imprinting to an ectopic locus, we targeted the repeats and DMD to the *Wnt1* locus. The *Wnt1* gene was chosen for two reasons. First, the gene is haplosufficient, so monoallelic expression induced by imprinting is not lethal [20]. Second, the expression of the gene from e9.5 to e11.5 is completely dependent upon a well-defined enhancer [21, 22]; therefore, the placement of methyl-sensitive enhancer-blocking sequences between the promoter and enhancer could plausibly control *Wnt1* expression. The allele constructed, shown in Fig. 1, positioned the ICR 3′ of the *Wnt1* transcribed domain and 5′ of the enhancer, which lies 3′ of the gene body. The ICR was placed in the same orientation relative to the enhancer and promoter that it assumes at *Rasgrf1*. The vector was then linearized and electroporated into ES cells, which were subjected to G418 selection; and homologous recombination was verified by both Southern blot and PCR (Additional file 2). Because the neomycin resistance cassette, with its high GC enhancer and promoter, could alter regulation of the *Wnt1* locus, we removed it using a FLPe expression vector prior to ES cell injections. Chimeras prepared by blastocyst injection transmitted the mutation through the germline. We refer to the allele as *Wnt1DR* (DR) indicating the presence of the DMD and the repeats at *Wnt1*.

**Fig. 2:**
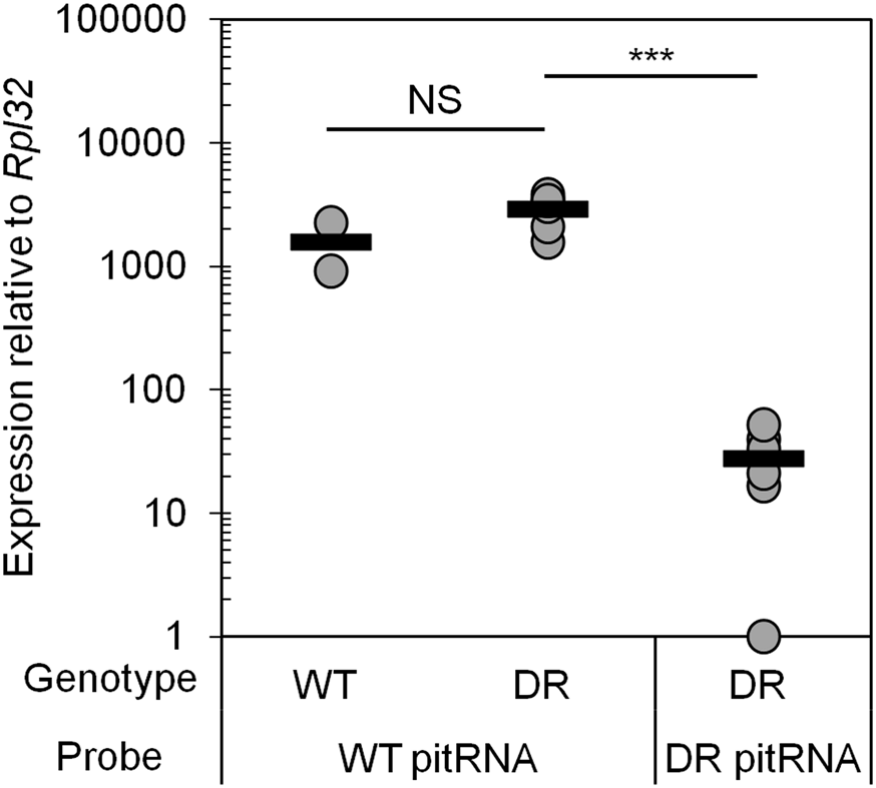
pitRNA expression from the *Wnt1DR* allele is significantly lower than the endogenous allele. Allele-specific qRT-PCR for the *Rasgrf1*-derived pitRNA (WT pitRNA) or the *Wnt1DR*-derived pitRNA (DR pitRNA) using 6 +/DR animals or 2 WT animals. Three *asterisks* represent p < 0.001 using a two-tailed *t* test, NS signifies not significant, and black horizontal lines indicate the average of relevant samples

Since pitRNA expression is necessary for proper DMD methylation in the male germline, we analyzed the e16.5 testis of heterozygous mutant animals. Allele-specific qRT-PCR after paternal transmission revealed that pitRNA expression from the *Wnt1DR* allele is less than 2 % that of the endogenous allele within the same animals (Fig. 2). The *Wnt1DR* allele did not affect expression levels at the endogenous allele, as no significant difference could be found in wild-type pitRNA expression between animals that did or did not carry the allele.

**Fig. 3:**
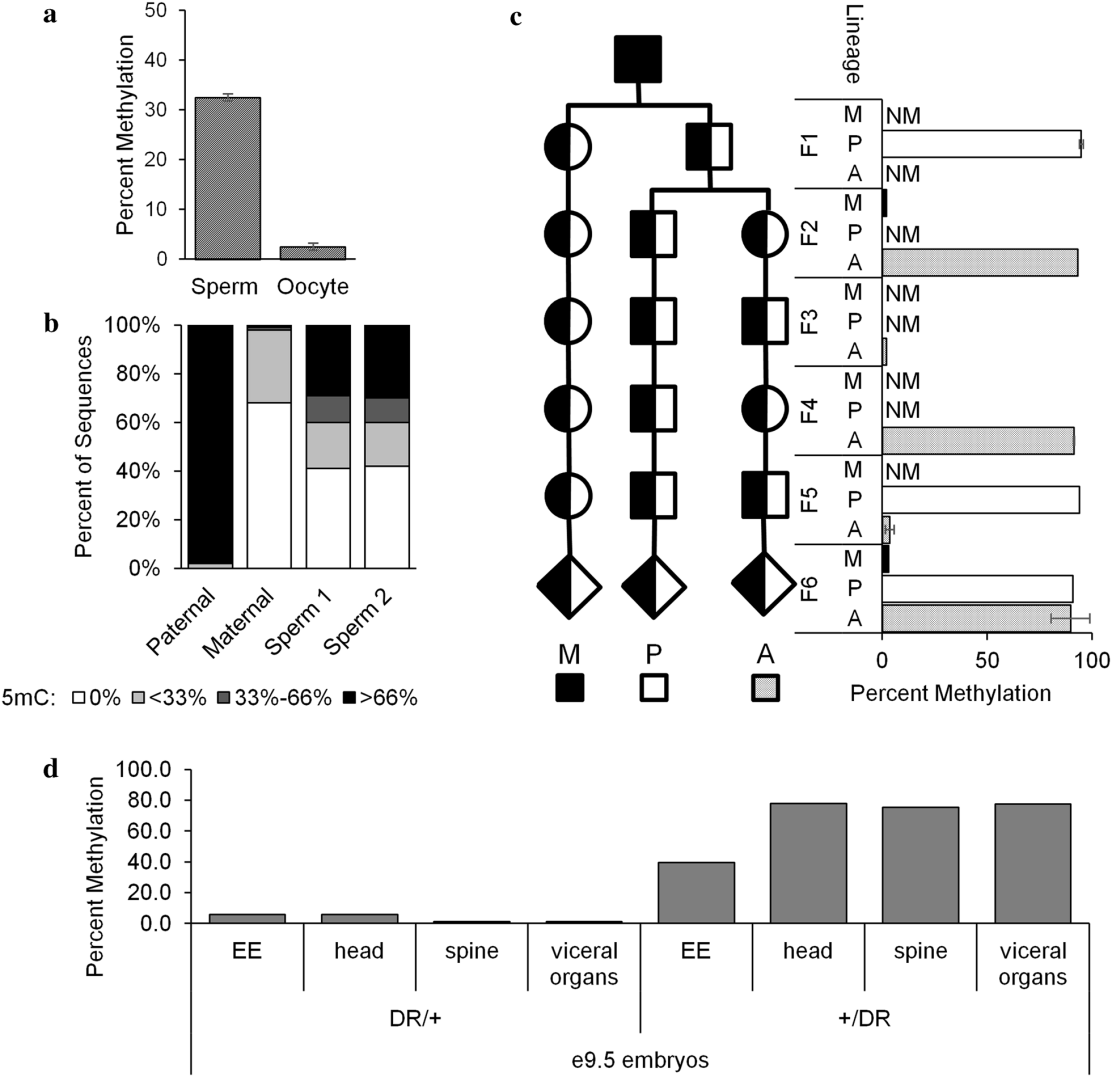
The *Wnt1DR* allele recapitulates partial DNA germline and complete somatic imprinted DNA methylation patterns. **a** Methylation levels at *Wnt1DR* in two DR/DR sperm samples and two pools of fifty DR oocytes as assayed by bisulfite sequencing. **b** Distribution of methylated CpGs within a paternal lineage F6 animal from (**c**), a maternal lineage F6 animal from (**c**), and the two sperm samples from (**a**). **c** *Left* mouse pedigrees with maternal (M), paternal (P), or alternating (A) transmission of *Wnt1DR*. *Right* DNA methylation levels, as assayed by bisulfite sequencing, using one to four animals sampled from the pedigrees. *Circles*, females; *squares*, males; *diamonds*, either sex; *half-filled shapes*, heterozygous animals; *NM* not measured. **d** High-throughput bisulfite analysis of extra embryonic (EE) tissue, head, spine, and internal organs of e9.5 embryos from either maternal (DR/+) or paternal (+/DR) transmission

We then set up pedigrees to fulfill three goals: first, to maintain the allele for six generations of passage through the female lineage; second, to maintain the allele for six generations of passage through the male lineage; and third, to alternate transmission through maternal and paternal lineages (Fig. 3c). Analysis of these pedigrees would reveal whether the *Wnt1DR* allele establishes and maintains the unmethylated state upon maternal transmission, the methylated state upon paternal transmission, and is properly reprogrammed upon each passage through the opposite sex’s germline. To increase throughput for the methylation analysis, we developed a protocol for ligating TruSeq adapters onto the bisulfite PCR products for next-generation sequencing. Compared to the standard bisulfite analysis pipeline of cloning followed by Sanger sequencing, this new workflow yielded, on a per sample basis, vastly increased read counts (Additional file 1), decreased cost, and decreased hands-on effort.

To investigate establishment of DNA methylation at the *Wnt1DR* allele, we analyzed sperm and oocytes from mutant animals and observed significantly higher 5mC in sperm compared to oocytes (Fig. 3a). Interestingly, at the *Wnt1DR* allele overall sperm methylation is 30 %, much lower than the 100 % methylation levels seen at the endogenous *Rasgrf1* locus. Methylation in sperm was not evenly distributed, with 41 % of the reads containing no 5mC, 29 % of the reads showing 66 % 5mC or higher, and the remaining reads displaying intermediate levels (Fig. 3b and Additional file 2). We infer that incomplete establishment of methylation in sperm is due to significantly reduced expression of pitRNA (Fig. 2).

Analysis of methylation maintenance at the *Wnt1DR* mutant allele in adult tail DNA showed full recapitulation of both patterns of maternal and paternal inheritance seen at the wild-type *Rasgrf1* ICR. The *Wnt1DR* allele preserved its unmethylated state in the soma during five generations of passage through the female germline (Fig. 3c). Transmission of the *Wnt1DR* allele through the male germline, despite low pitRNA expression and incompletely established germline methylation, displayed a fully methylated state in the soma throughout six generations of passage (Fig. 3c). Analysis of the alternating lineage pedigree demonstrated that the methylation profile could be faithfully reset at multiple different generations (Fig. 3c). These data demonstrate that the ~3-kb section of the ICR is sufficient for partial establishment, and full maintenance of the 5mC imprint. We extended these somatic DNA analyses, querying methylation states of DNAs from extra embryonic tissue, head, spine, and visceral organs taken from e9.5 embryos, the stage when the *Wnt1* locus is expressed. We observed comparable patterns of methylation seen in adult tail DNAs: The *Wnt1DR* allele was significantly more methylated after paternal transmission than maternal transmission in each tissue (Fig. 3d). However, the levels of methylation in embryonic tissues were consistently lower than levels found in adult tail DNAs. This is consistent with the possibility that in our system, methylation increases over developmental time. We did not assay methylation in preimplantation embryos and do not know whether the *Wnt1DR* allele resists global demethylation, as is typical for ICRs, or whether it undergoes demethylation along with the rest of the genome.

The abundance of unmethylated DNA in sperm, but its relative absence in adult somatic DNA after paternal transmission of the *Wnt1DR* allele (Fig. 2b), raised the possibility that unmethylated sperm may be less competent at fertilization and that adults analyzed were the result of fertilization by more competent and methylated sperm. To test this possibility, we isolated highly motile sperm from a sperm cell pellet by a swim-up assay and then compared the methylation state of the mobile sperm to that of sperm remaining in the cell pellet. Results showed that the mobile sperm were no more methylated than the sperm in the pellet (Additional file 3), indicating that unmethylated sperm were not at a motility disadvantage.

**Fig. 4:**
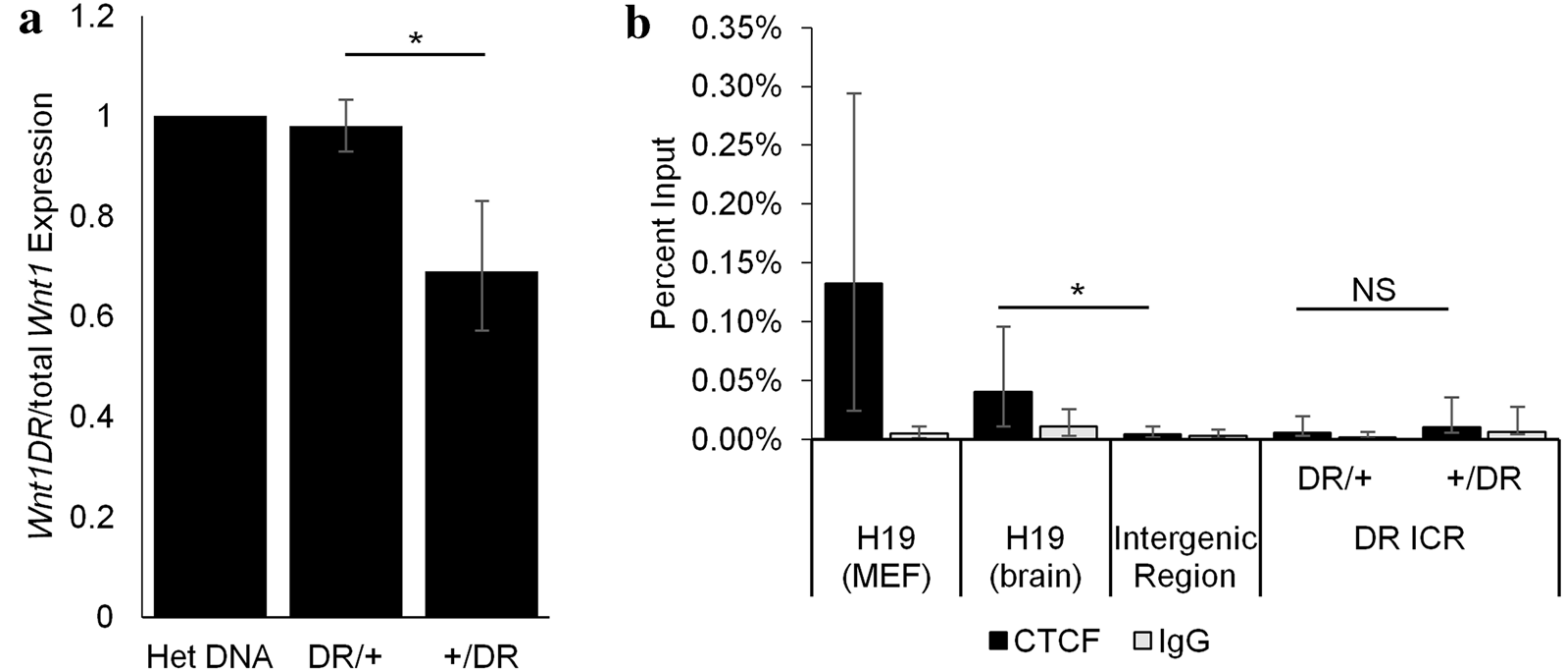
Maternal transmission of *Wnt1DR* allele does not cause silencing or CTCF binding. **a** Allele-specific qRT-PCR using e9.5 brains taken from four mice with maternal (DR/+) or paternal (+/DR) transmission of *Wnt1DR*. Data are presented as the ratio of *Wnt1DR* allele-specific expression to total *Wnt1* expression. Heterozygous DNA (Het DNA) was used as a calibrator for the 1:2 copy number of *Wnt1DR* to total *Wnt1*. *Asterisk* signifies p < 0.05 using a two-tailed *t* test. **b** CTCF ChIP. Chromatin from mouse embryonic fibroblasts (MEFs) or e9.5 brain (remaining samples) was subjected to ChIP using anti-CTCF or non-immune IgG. Purified DNAs were subjected to qPCR amplifying *H19* as positive control for MEF and e9.5 brain chromatin samples, an intergenic region containing no CTCF sites as a negative control for, and the *Wnt1DR* allele. Three biological replicates were used. *Asterisk* represents p < 0.05 using a two-tailed *t* test after accounting for multiple hypothesis testing using Bonferroni’s correction. *NS* not significant. *Error bars* denote standard deviation with error propagation

Somatic regulation of 5mC at the endogenous *Rasgrf1* DMD is required to control the methylation-sensitive binding of CTCF. Unmethylated sequences permit CTCF binding and enhancer-blocking function to silence transcription from the maternal allele [23]. We therefore assayed expression of *Wnt1* from our construct to determine whether the faithfully imprinted 5mC at *Wnt1DR* enabled allele-specific expression. Our design of the *Wnt1DR* allele included placement of a four-nucleotide polymorphism in the 3′ UTR to facilitate allele-specific qRT-PCR analysis. We found that maternal transmission of the allele did not repress *Wnt1* (Fig. 4a). Interestingly, we found that the paternal allele was slightly repressed, possibly due to the proximity of the *Wnt1* gene and its enhancer to the highly methylated ICR.

There are two explanations for the inability to recapitulate imprinted expression. First, CTCF might not be bound to our mutant construct, possibly because the binding sites within the ICR are weak and might need external sequences for proper recruitment. CTCF has been shown to work in pairs to change the three-dimensional structure of the DNA, so a second possibility might be that CTCF is correctly localized, but cannot function without interacting partner sites that can sequester the enhancer, as occurs at the globin locus control region [24]. To determine whether CTCF is bound to the unmethylated, maternally transmitted *Wnt1DR* allele, we performed ChIP-qPCR which revealed that while there was significant binding at the positive control (*H19* ICR) in both mouse embryonic fibroblasts (MEFs) and e9.5 brains, there is no significant CTCF binding to the negative control or to the *Wnt1DR* ICR (Fig. 4b) in e9.5 brains. In MEFs, the endogenous *Rasgrf1* ICR binds CTCF, whereas the ectopic *Wnt1DR* ICR does not (Additional file 4). Therefore, we conclude that the *Wnt1DR* allele either lacks CTCF-recruiting sequences that were present at the endogenous locus, that the novel chromosomal context at the *Wnt1* locus acts to negatively regulate CTCF binding, or that overall binding of CTCF in e9.5 brain is sufficiently low that imprinted expression of *Wnt1* is not possible, even if the required *cis*-acting elements for imprinting are present and functional.

## Discussion

Temporally controlled, tissue-specific targeting of DNA methylation to specific sequences is required for normal fertility and health in mammals. Very few systems have identified sequences which are sufficient to recruit epigenetic effectors of 5mC, and even those sequences appear to act by a variety of mechanisms. Here, we investigate the *Rasgrf1* imprinted region and define a 3-kb portion of the ICR that is sufficient to recapitulate many features of *Rasgrf1* imprinting when exported to the ectopic *Wnt1* locus.

In oocytes, only 2.5 % of the CpGs in 19,418 bisulfite reads analyzed harbored 5mC at *Wnt1DR*, consistent with the lack of 5mC in oocytes reported at *Rasgrf1*. Similarly, upon maternal transmission of *Wnt1DR*, only 2.6 % of the CpGs in 65,497 bisulfite reads were methylated, also consistent with the methylation state at the maternal copy of *Rasgrf1.*

The pitRNA, whose expression in e16.5 testes is necessary in *cis* for imprinted DNA methylation at *Rasgrf1* [16, 17], is expressed from *Wnt1DR*. However, its expression level is only 2 % that seen from *Rasgrf1*. This weak expression may be due to undefined sequences at *Rasgrf1* that are necessary for full expression, but are absent from the *Wnt1DR* allele. Alternatively, the necessary sequences may be present, but the chromatin context at *Wnt1* may not be permissive for their full activity. A previously published allele [17] contained fewer sequences from the endogenous ICR relative to the *Wnt1DR* construct, but still established robust sperm methylation, arguing for the latter hypothesis. Despite the low level of pitRNA expression, it is possible that it was sufficient to establish methylation in the male germline at an average of 30 % of the CpGs assayed at *Wnt1DR*, based on analysis of 6632 bisulfite sequencing reads from sperm DNA. Of those reads, 41 % showed no *Wnt1DR* methylation, and an equal percentage showed methylation levels between 33 and 100 %. A similar pattern was revealed upon separation of sperm by motility, eliminating the possibility that sperm were unmethylated merely because of fundamental defects affecting their maturation to the motile stage. An alternative interpretation is that the pitRNA is dispensable for methylation at *Wnt1*.

Intriguingly, despite the abundance of unmethylated and partially methylated sperm, somatic methylation was nearly complete in 11 adult mice tested with a paternally transmitted *Wnt1DR* allele; 92 % of CpGs assayed in 54,923 bisulfite reads were methylated. This result suggests either that the modest levels of pitRNA expression and the partial establishment of male germline methylation are sufficient for full somatic methylation, or that germline methylation is not necessary for full somatic methylation. In e9.5 embryonic tissues of mice with a paternally transmitted *Wnt1DR* allele, 75 % of CpGs assayed in 22,162 bisulfite reads were methylated. When compared to the 92 % rate in adult tissues, one might infer that acquisition of methylation is progressive after fertilization, rather than depending upon preexisting methylation in sperm. We cannot know both the methylation state of a given sperm, and the somatic methylation of the mouse resulting from its fertilization. It is possible that the 11 adult progeny assayed after paternal transmission of *Wnt1DR* arose only from the 59 % of sperm that were richly methylated. However, assuming that fertilization fitness among sperm does not depend upon *Wnt1DR* methylation state, there is less than a 0.3 % probability that this is the case. If germline methylation is necessary for somatic methylation, then in animals arising from fertilization by sperm lacking methylation on the *Wnt1DR* DMD, the somatic methylation may have occurred by methylation spreading from the repeats, sperm-transmitted pitRNA, or another unknown mechanism. Because of their repetitive nature, the repeats are difficult to characterize for methylation status; however, they are unlikely to exhibit a methylation profile which differs from the DMD because our assay spans the area between the repeats and pitRNA sites 1 and 2—the sequences which recruit DNA methylation. Sperm-transmitted RNAs are also unlikely, as piRNAs appear to be depleted in mature spermatozoa [25, 26]. It is formally possible, however, that a limited number of piRNAs and their effector proteins are still active after fertilization.

Allele-specific histone modifications like H3K9me2 [27], H3R3me2 [28], H3K9me3 [29], or H4K20me2 [29], rather than 5mC, could represent the primary imprinting mark, which subsequently enables somatic DNA methylation at *Wnt1*. Indeed, H3K9me2 is present on the *Rasgrf1* ICR and has already been shown to be vital for imprinting maintenance; it recruits PGC7 which subsequently protects against Tet-mediated demethylation of the paternal allele after fertilization [27, 30]. This mark could also be sufficient to recruit de novo DNA methylation to the paternal allele after fertilization in the absence of a preexisting methylation. Caution must be advised when interpreting histone immunoprecipitation experiments in sperm; however, as histones are switched for protamines in post-meiotic spermatids, leading to low total histone occupancy and inflated signal from rare epigenetic marks, compared to tissues lacking protamine substitutions. Analysis of the *Rasgrf1* ICR in particular revealed a depletion of nucleosomes, potentially complicating any interpretation [31]. Our finding that somatic methylation of *Wnt1DR* may not depend on prior methylation establishment is consistent with reports of knock-in alleles of the *H19* ICR at *Afp* [11] and *CD3* [14], as well as other models [12, 13] which also displayed incomplete germline methylation followed by complete somatic methylation. It is also consistent with the work examining methylation acquisition of *Igf2r* ICR sequences, which was done by injecting DNA into post-fertilization embryos [7], not into oocytes where DNA methylation establishment normally occurs [32]. Finally, the possibility that somatic 5mC can arise in the absence of previously established 5mC in sperm is consistent with our previous findings that sperm methylation is not sufficient for somatic maintenance of that mark [18].

A previous transgenic model to test the sufficiency of the *Rasgrf1* ICR for control of imprinted DNA methylation failed to exhibit reversible somatic methylation upon sequential passage through the male and female germlines [17]. Analysis of that model was confounded by persistent methylation of a neo reporter cassette. In contrast, the *Wnt1DR* allele exhibited the expected methylation reversal across six generations, demonstrating the sufficiency for somatic methylation imprinting of the tested sequences.

Although somatic methylation of the *Wnt1DR* allele faithfully recapitulated what was observed on the endogenous *Rasgrf1* allele, this was not sufficient to impart imprinted expression to *Wnt1*. Imprinted expression at *Rasgrf1* requires silencing of the maternal allele by CTCF binding to the DMD, which limits activity of the maternal promoter. Methylation at the paternal allele prevents CTCF binding, and enhancer–promoter interactions are unrestricted. The binding of CTCF to *Wnt1DR* was undetectable above background, which might account for the failure of the unmethylated maternal *Wnt1DR* allele to be silenced. Lack of CTCF binding is likely due to its complex role in mediating the formation of three-dimensional chromatin structures with multiple interacting CTCF-binding sites [33–36]. The local chromosomal context at *Wnt1* may lack appropriate partner sites for the *Wnt1DR* ICR and therefore discourage this complex binding. Alternatively, if CTCF-binding activity is limited in e9.5 brain, this might be sufficient to explain lack of imprinted expression at *Wnt1DR.*

## Conclusions

We have identified sequences that are sufficient for partial germline, and full somatic 5mC imprinting at an ectopic locus. *Rasgrf1* is one of the few loci whose *cis*-acting sequences have been so narrowly defined. Discordance between the levels of *Wnt1DR* methylation in sperm and somatic tissue after paternal transmission is consistent with prior findings that post-fertilization methylation is not merely a maintenance of gametic methylation, indicating the existence of distinct post-fertilization methylation mechanisms.

## Additional files

10.1186/s13072-016-0094-0 Breakdown of MiSeq methylation data. Libraries were analyzed in two separate runs. In the first run (*), all 6 libraries were placed into <1% of a lane while in the second run (†), each library received ~1% of a lane. Files from the second run were prohibitively large and were therefore truncated to 10MB (~38,653 sequences) before QUMA analysis. PHRED filter: 90% of the read >20. QUMA filter: >98% identity, <10 mismatches, >95% conversion, <5 unconverted CpHs. Supernatant (sup.); animal from Figure 3C maternal lineage (M); animal from Figure 3C paternal lineage (P); animal from Figure 3C alternating lineage (A).
10.1186/s13072-016-0094-0 Confirmation of correct integration of Wnt1DR construct. A) Schematic of Southern probes and internal PCR used in panels B and C. B) Southern blots on NdeI digested ES cell DNA were probed with either an external 5’ probe or an external 3’ probe with the expectation of a wild type 11.74kb band and a mutant 9.76kb and 7.47kb, respectively. Example of positives in lane 5 of both gels. C) Internal PCR as depicted in panel A was used to follow construct transmission. Rpl32 was used as a loading control.
10.1186/s13072-016-0094-0 Distribution of methylation levels in sperm DNA. Rank order analysis (A) and bar charts (B) were used to give a more detailed view of the distribution of methylation levels within Sperm 1 and Sperm 2 samples described in Figure 3B. Sperm 3 and Sperm 4 are derived from swim up assays that separate motile (sup.) from non-motile (pellet) sperm.
10.1186/s13072-016-0094-0 CTCF binding in MEF cells. CTCF ChIP was performed as in Figure 4, using MEF cells as a chromatin source. The endogenous Rasgrf1 ICR (WT ICR), shows significant CTCF binding; whereas the ectopic Wnt1DR ICR, shows no significant binding.

## Abbreviations

ICR: imprinting control region
5mC: cytosine methylation
ADS: allele-discriminating sequence
DNS: de novo methylation signal
DMD: differentially methylated domain
pitRNA: piRNA-targeted RNA
neo: neomycin selection cassette
ChIP: chromatin immunoprecipitation
qRT-PCR: quantitative reverse transcription-polymerase chain reaction
H3K9me2: histone three lysine nine dimethylation
H3R3me2: histone three arginine three dimethylation
H3K9me3: histone three lysine nine trimethylation
H4K20me2: histone four lysine twenty demethylation
