# Supplementary material for: Imprinted DNA methylation reconstituted at a non-imprinted locus
Source: Epigenetics Chromatin. 2016 Sep 22;9:41. doi: 10.1186/s13072-016-0094-0 (PMC5034545; doi:10.1186/s13072-016-0094-0)
Supplement: Supplementary file 1 — 10.1186/s13072-016-0094-0 Breakdown of MiSeq methylation data. Libraries were analyzed in two separate runs. In the first run (*), all 6 libraries were placed into <1% of a lane while in the second run (†), each library received ~1% of a lane. Files from the second run were prohibitively large and were therefore truncated to 10MB (~38,653 sequences) before QUMA analysis. PHRED filter: 90% of the read >20. QUMA filter: >98% identity, <10 mismatches, >95% conversion, <5 unconverted CpHs. Supernatant (sup.); animal from Figure 3C maternal lineage (M); animal from Figure 3C paternal lineage (P); animal from Figure 3C alternating lineage (A). [file 13072_2016_94_MOESM1_ESM.pdf]

Breakdown of MiSeq methylation data

Animal number	Generation and lineage	Total Miseq sequences	Sequences passing PHRED filter	Sequences passing QUMA thresholds
31092*	F1 P	17,518	9,489	4,064
31150†	F1 P	239,206	151,916	3,406
31153†	F1 P	283,512	165,705	2,806
30999†	F1 P	224,213	67,996	2,700
31071*	F2 M	26,941	14,815	7,622
31075*	F2 M	26,334	15,844	9,397
31078†	F2 A	279,920	197,280	2,915
31289†	F3 A	205,607	147,078	3,527
31544†	F3 A	288,652	170,890	1,905
31508†	F4 A	707,055	306,648	7,309
31819†	F4 A	663,137	266,451	11,234
32415†	F5 A	287,049	213,814	3,743
32181†	F5 A	260,495	180,937	1,738
32178†	F5 A	238,913	87,920	3,795
32803*	F6 M	35,683	21,547	13,480
32804*	F6 M	41,468	25,831	12,543
32802*	F6 P	27,718	17,099	7,513
32339†	F6 A	227,530	161,457	3,581
32529†	F6 A	141,201	107,655	6,315
32530†	F6 A	200,173	54,830	3,080
35084†	Sperm	280,699	92,637	2,563
35090†	Sperm	184,452	125,431	4,069
35669 pellet†	Sperm	231,561	175,028	26,901
35669 sup.†	Sperm	155,302	119,684	15,679
35670 pellet†	Sperm	312,711	243,539	25,603
35670 sup.†	Sperm	212,255	167,219	16,270
Oocyte pool 1†	Oocyte	215,808	126,407	1,870
Oocyte pool 2†	Oocyte	341,780	41,574	17,548
E9.5 EE†	+/DR	593,195	490,141	8,735
E9.5 head†	+/DR	579,546	485,625	8,210
E9.5 spine†	+/DR	537,237	451,830	8,521
E9.5 organs†	+/DR	642,148	482,669	7,556
E9.5 EE†	DR/+	859,350	308,544	8,798
E9.5 head†	DR/+	679,635	355,402	7,033
E9.5 spine†	DR/+	461,931	350,100	7,898
E9.5 organs†	DR/+	486,448	393,705	7,231
