# Supplementary figures and images for: Imprinted DNA methylation reconstituted at a non-imprinted locus
Source: Epigenetics Chromatin. 2016 Sep 22;9:41. doi: 10.1186/s13072-016-0094-0 (PMC5034545; doi:10.1186/s13072-016-0094-0)

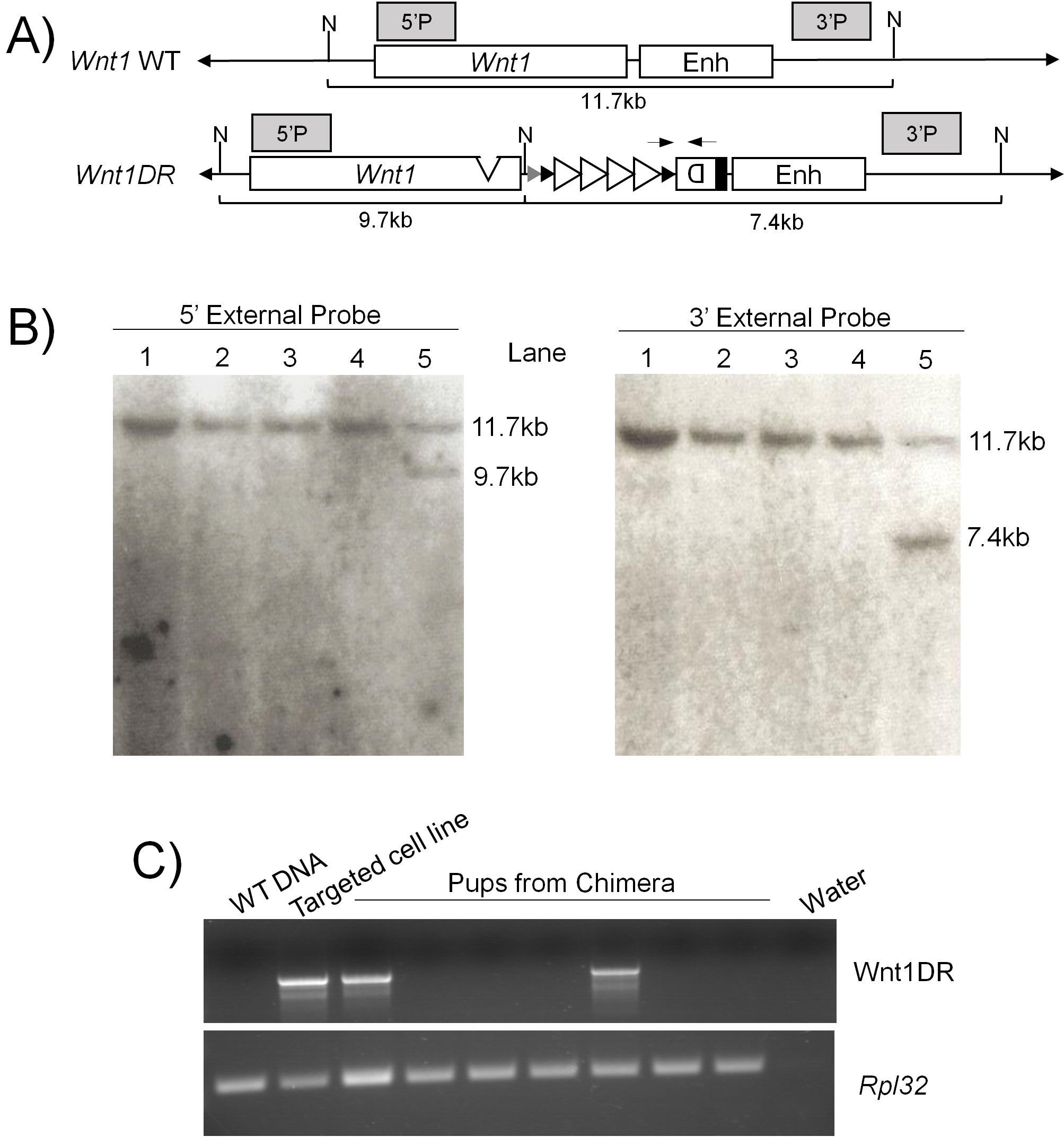

Supplement: Supplementary file 2 — 10.1186/s13072-016-0094-0 Confirmation of correct integration of Wnt1DR construct. A) Schematic of Southern probes and internal PCR used in panels B and C. B) Southern blots on NdeI digested ES cell DNA were probed with either an external 5’ probe or an external 3’ probe with the expectation of a wild type 11.74kb band and a mutant 9.76kb and 7.47kb, respectively. Example of positives in lane 5 of both gels. C) Internal PCR as depicted in panel A was used to follow construct transmission. Rpl32 was used as a loading control. [file 13072_2016_94_MOESM2_ESM.tif]

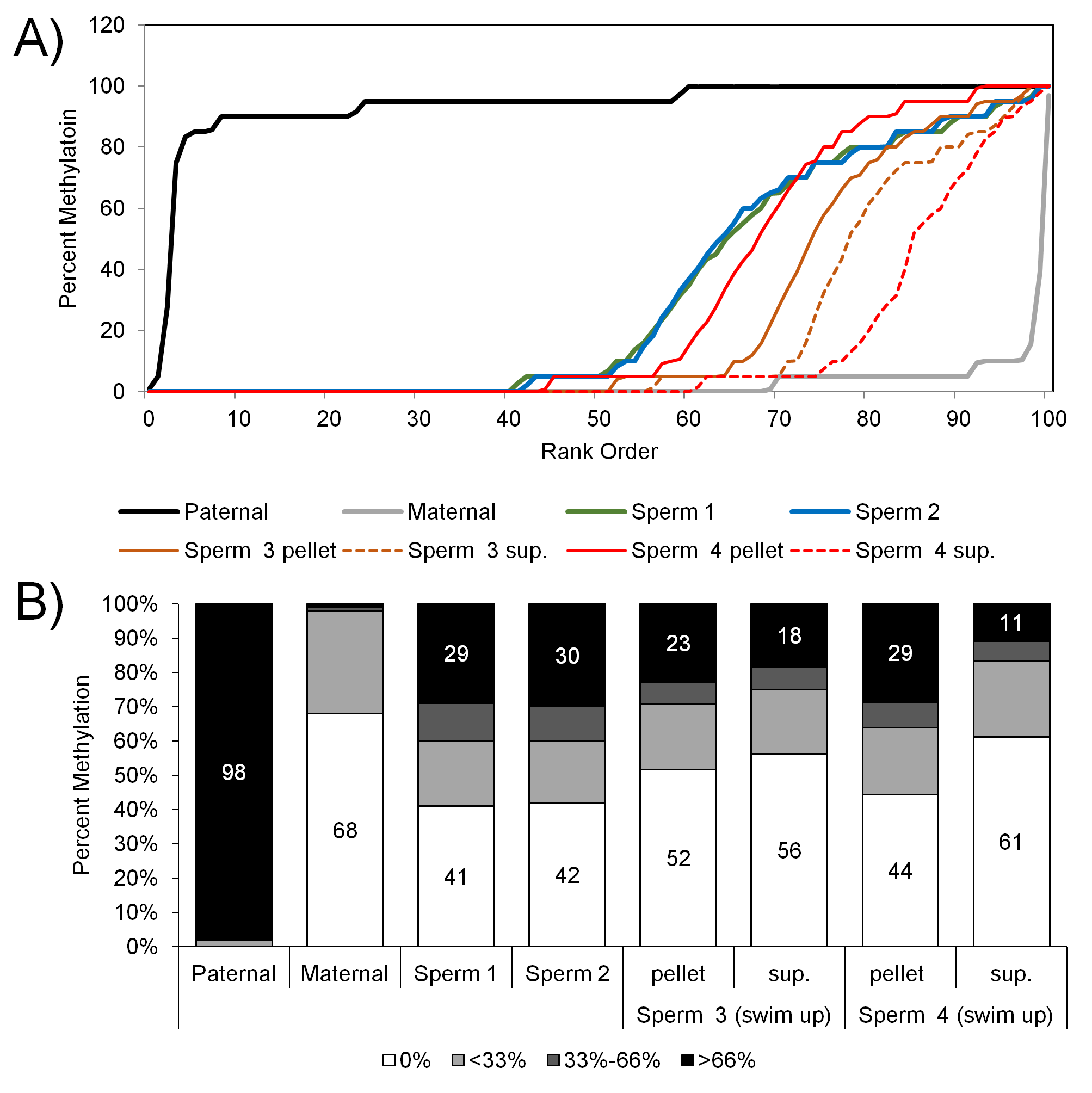

Supplement: Supplementary file 3 — 10.1186/s13072-016-0094-0 Distribution of methylation levels in sperm DNA. Rank order analysis (A) and bar charts (B) were used to give a more detailed view of the distribution of methylation levels within Sperm 1 and Sperm 2 samples described in Figure 3B. Sperm 3 and Sperm 4 are derived from swim up assays that separate motile (sup.) from non-motile (pellet) sperm. [file 13072_2016_94_MOESM3_ESM.tif]

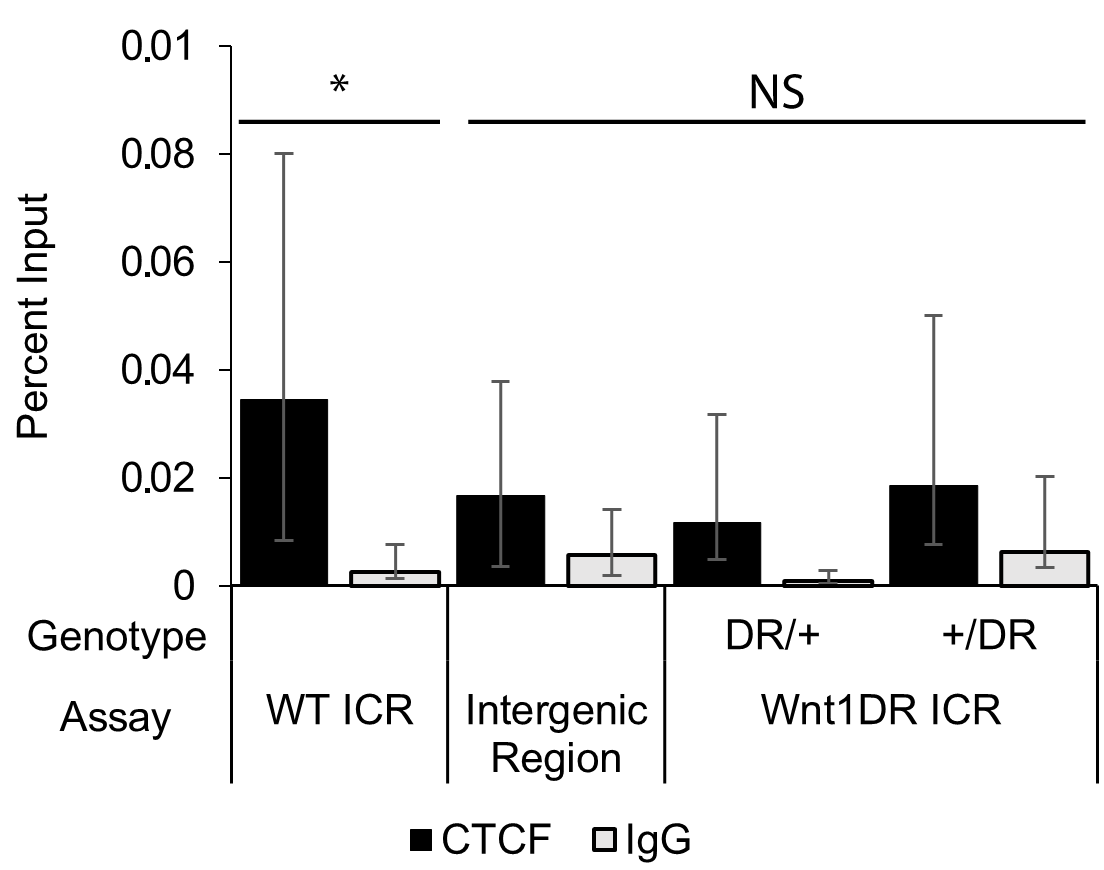

Supplement: Supplementary file 4 — 10.1186/s13072-016-0094-0 CTCF binding in MEF cells. CTCF ChIP was performed as in Figure 4, using MEF cells as a chromatin source. The endogenous Rasgrf1 ICR (WT ICR), shows significant CTCF binding; whereas the ectopic Wnt1DR ICR, shows no significant binding. [file 13072_2016_94_MOESM4_ESM.tif]
